# Multiple strategies and cues define baleen whale migration

**DOI:** 10.1098/rspb.2026.0029

**Published:** 2026-10-14

**Authors:** Leena Riekkola, Xuelei Zhang, Alice Della Penna, Virginia L. Andrews-Goff, Gabriela Duran, Rochelle L. Constantine, Simon Childerhouse, Fredrik Christiansen, Rosalind Cole, Kim Goetz, Robert Harcourt, David Lundquist, Catherine E. Meyer, Mike Ogle, Esther Stuck, Alexandre N. Zerbini, Emma L. Carroll

**Affiliations:** ^1^ School of Biological Sciences, The University of Auckland, Auckland 1010, New Zealand; ^2^ Institute of Marine Science, The University of Auckland, Auckland 1010, New Zealand; ^3^ Australian Marine Mammal Centre, Australian Antarctic Division, Kingston, Tasmania 7050, Australia; ^4^ Blue Planet Marine, Nelson 7050, New Zealand; ^5^ Marine Mammal Research, Department of Ecoscience, Aarhus Universitet, 4000 Roskilde, Denmark; ^6^ New Zealand Department of Conservation, Wellington, 6011, New Zealand; ^7^ Marine Mammal Laboratory, NOAA Fisheries Alaska Fisheries Science Center, Seattle, WA 98115, USA; ^8^ School of Natural Sciences, Macquarie University, Sydney, New South Wales 2109, Australia; ^9^ Cooperative Institute for Climate, Ocean & Ecosystem Studies, University of Washington, Seattle, WA, USA; ^10^ Marine Ecology and Telemetry Research, Seabeck, WA, USA; ^11^ Instituto Aqualie, Juiz de Fora, MG, Brazil; ^12^ Whale Unit, Mammal Research Institute, University of Pretoria, South Africa

**Keywords:** migratory cues, migratory departure timing, migratory strategy, navigation, satellite telemetry, southern right whale, *Eubalaena australis*

## Abstract

Migration is a do-or-die life-history strategy, yet factors shaping it remain poorly studied in many marine taxa, as direct observations are difficult. Building on animal migration theory, we used satellite tracking data from 21 southern right whales tagged in Aotearoa New Zealand to investigate migratory cues, timing and routes. Whales followed either a direct westward route or a longer, indirect route via higher latitudes when migrating from their wintering grounds to summer feeding grounds. Departure timing from wintering grounds was the only significant predictor of route choice, independent of sex, age class, body length or condition. Early-departing whales were more likely to follow the longer route, taking more days to migrate, yet they arrived at shared feeding grounds at similar times to whales using the direct route. These contrasting behaviours are hypothesized to reflect trade-offs between time- and energy-minimization strategies. During migration, whales may use multiple external cues to navigate, including gradients associated with ocean currents, bathymetry and magnetic inclination. The availability of two distinct migratory routes, combined with individual flexibility in route choice, may enhance population resilience in a changing ocean.

## 1. Introduction

Migration is a widespread evolutionary strategy found across invertebrates and all major vertebrate lineages in aquatic, terrestrial and aerial environments [1,2]. It is a behavioural adaptation to seasonal variability in resources, habitats, predation and competition, driving directed movements between geographically separated sites that fulfil critical life-history needs, such as reproduction or feeding [1,2].

Migration is shaped by intrinsic and extrinsic (environmental) factors that influence when, where and how animals move. For example, migratory restlessness, which can trigger departure, is intrinsic to many animals [3,4], while extrinsic cues, such as vegetation green-up, can shape the timing of herbivore migration (i.e. ‘surfing the green wave’) [5,6]. Once underway, the exact path can be honed by factors such as individual traits, social learning and prevailing environmental conditions. For example, age or experience can influence aspects of migration, e.g. route choice, which individuals may learn from personal experience or conspecifics [7–9]. Environmental factors, such as wind patterns, can further shape the specific paths animals take or their use of stopover sites during migration [10,11]. Successful migration also depends on navigation, hypothesized to use a combination of strategies at different spatial scales. Long-distance travel may use ‘map and compass’ systems, where animals determine their position relative to a goal (map) and then orient and maintain direction towards it (compass) [12,13]. The compass component can be based on celestial (e.g. stars and Sun) or geomagnetic (e.g. inclination) cues [13,14]. At smaller spatial scales, animals use local environmental indicators, for example visual landmarks or olfactory cues, to navigate [15–17].

Through these mechanisms, migratory animals may optimize their journeys by balancing time- or energy-minimization strategies. Time-minimization involves moving faster, potentially by-passing stopover sites, to complete migration faster, even at the expense of higher energetic costs [18,19]. By contrast, an energy-minimization strategy involves moving slower (or at/near the optimal cost-of-transport speed), using stopover sites to refuel and/or exploiting favourable winds or currents, thereby minimizing either the energetic cost of transport or total energy expenditure across the migratory journey [19,20]. These contrasting strategies provide a useful framework for investigating migratory behaviour in poorly studied taxa.

While the factors influencing migration are well studied in some taxa, particularly birds [9,10,12,13], less is known in other animals, such as whales [17,21,22]. Many baleen whale species undertake annual migrations between summer feeding grounds and winter breeding/calving grounds [23,24]. Intrinsic cues, including body condition, sex and reproductive status, are hypothesized to influence migratory timing, routes and travel speed [25–29]. For the great whales, individuals in poorer body condition, or females migrating with calves, may face increased energetic constraints and favour energy-minimizing strategies through direct or slower routes (e.g. [30]). In contrast, individuals in better condition, with larger energy reserves, may delay departure from breeding grounds and travel faster, adopting a time-minimizing strategy (as seen in birds, e.g. [31,32]). Environmental factors, such as day length or increasing sea ice, have also been linked to whale departure from breeding or Antarctic feeding grounds, respectively [27,33]. For navigation, whales may use a range of environmental cues across different spatial scales. For example, geomagnetic features and oceanographic structures could provide directional or positional information for long-distance navigation, through, e.g. magnetic inclination [17,22] or temperature and salinity gradients in frontal systems (reviewed in [17,34]).

Here, we investigate how intrinsic and extrinsic factors shape migration in southern right whales (*Eubalaena australis*; SRWs). This species migrates seasonally between productive mid- to high-latitude summer feeding grounds and sheltered, low- to mid-latitude winter breeding/socializing/calving (hereafter ‘wintering’) grounds [35–37]. Historical knowledge of SRW migration came from wintering-ground monitoring with aerial, land-based or genetic methods [38–41]. From such studies, we know that SRWs show fidelity to maternally inherited migratory destinations [40,41]. Extrinsic factors, e.g. day length, and intrinsic factors, e.g. calf size, can also influence SRW migratory departure timing [27,42], patterns seen in other baleen whales, like humpback (*Megaptera novaeangliae*), North Atlantic right (*Eubalaena glacialis*) and fin (*Balaenoptera physalus*) whales [25,29,43,44].

Regardless of the cues driving SRW migration, little was known about their migratory behaviour or precise routes until the advent of satellite telemetry. Bio-logging has enabled tracking of whales across ocean basins, revealing new information on migratory movements and behaviour [e.g. 37,45]. Understanding migration is essential for predicting how this critical life-history trait may be affected by human impacts, such as shipping, and changing climatic conditions. Here, we integrate satellite tracking, body condition, life-history and environmental data to investigate SRW migration, including departure timing, migratory routes and potential environmental cues, in the ‘Threatened-Nationally Increasing’ [46] Aotearoa New Zealand (NZ) population. This population winters at the subantarctic Maungahuka Auckland Islands (AI) (between approx. May and September). As individuals migrate to shared summer feeding grounds south of Australia (approx. October–February) [37], this population provides a unique opportunity to study variation in migration routes among years and individuals. Based on migratory theory, SRWs may employ alternative strategies that balance time and energy constraints. Specifically, we expect that (i) individuals departing later from the breeding grounds will be more likely to adopt shorter, more direct routes and/or travel faster to the feeding grounds (time-minimization strategy), (ii) individuals with greater energetic constraints (e.g. smaller body size or poorer body condition) will favour more direct routes to the feeding grounds (energy-minimization strategy), and (iii) environmental features (e.g. oceanographic structure and geomagnetic cues) will be associated with movement patterns during migration.

## 2. Methods

### (a) Study region and data collection

SRWs (*n* = 25) were satellite-tagged in AI, NZ in August 2020, June/July 2021 and July 2022 [37,47]. Briefly, consolidated (Type C) [48,49] Wildlife Computers’ (Redmond, Washington, USA) Argos satellite tags were deployed on mature whales in good body condition [50]. Tags were programmed to uplink to satellites optimizing duty cycling for battery life and Argos satellite coverage across the Indo-Pacific region [37].

Skin biopsy samples were collected at the time of tagging using a crossbow or a modified veterinary capture device firing a small, sterile dart [51]. DNA was extracted from each sample, and DNA profiles comprising genetically identified sex, mitochondrial haplotype and multi-locus microsatellite genotype (up to 17 loci) were constructed [51]. Individuals accompanied by a calf (less than two-thirds the body length of the accompanying adult) were considered cows [51].

Photogrammetry images were obtained using unmanned aerial vehicles (UAVs) and total body length and body widths were extracted at 5% intervals following published methods [52–54]. Body volume was then calculated using a segmented elliptical model [55], based on each whale’s length, width and height measurements. We estimated body condition index (BCI) for each whale as the proportional difference between observed (measured) and expected body volume, with positive BCI values indicating greater-than-expected body volume for a given length and negative values indicating poorer condition [53,56]. For further details see supplementary Material.

### (b) Southern right whale migratory behaviour

We corrected for location error in the satellite tracking data through state-space models (SSMs) using the R (v. 4.4.3) [57] package *aniMotum* (v. 1.2-06) [58]. We estimated move persistence values at observed location time stamps using the *fit_mpm* function, which evaluates the autocorrelation between speed and directionality [58]. Following [37], move persistence values were classified as area-restricted search (ARS; indicative of foraging, socializing or resting) or travelling behaviour.

To analyse different portions of SRW migration tracks, we characterized tracking data into outbound (from wintering grounds to feeding grounds) and inbound (from feeding grounds to wintering grounds) sections. The start of outbound migration was defined as the point at which individuals began sustained, directed movement away from the AI wintering ground, reflected by relatively higher move persistence values, at least 20 km from the islands. This threshold was based on visual inspection of tracks to exclude near-shore movements while avoiding overestimation of departure timing, and to maintain consistency with previous work [37]. Outbound migration was considered complete when individuals arrived at presumed summer feeding grounds [37], indicated by a minimum of five consecutive ARS locations or a visually tortuous track used as a qualitative cue. We recorded the date of both wintering-ground departure and feeding-ground arrival for each whale. Inbound migration back to the wintering ground was classified visually and defined as relatively direct movement from the feeding grounds eastward, towards the AI.

We categorized outbound migration routes as ‘L-shaped’ or west/northwest (W/NW) routes based on visual examination of route shape and whether individuals crossed 55°S. The W/NW route followed a relatively straight west or northwest path from the AI, remaining north of 55°S, while the L-shaped route initially headed south/southwest, crossing 55°S, before turning northwest towards Australia. For whales using the L-shaped route, each location point was visually assigned a turn phase (‘before’, ‘during’ or ‘after’ the turn). The intermediate phase (‘during’) was included since the turn was often gradual rather than abrupt, making it unsuitable to classify some tracks solely as ‘before’ or ‘after’ (supplementary material, figure S1). We calculated bearing between consecutive locations using the *bearing* function in the *geosphere* R package (v. 1.5-10) [59]. Bearing values, ranging from –180° to +180°, were converted to standard compass bearings (0–360°) where 0° represents true north and 180° south. We plotted resulting compass bearing distributions for the three location categories (‘before’, ‘during’ and ‘after’).

The arrival of SRWs at their shared feeding grounds coincides with the period of peak productivity [37]. We therefore tested whether the different migratory routes reflect time- and energy-minimization strategies. Specifically, we predicted that individuals departing later adopt shorter, more direct routes and/or travel faster to compensate for reduced time available for migration (time-minimization strategy). We then evaluated whether individual traits, such as body length (proxy for age), body condition, sex or reproductive status, influenced route choice, with the expectation that individuals with higher energetic demands or constraints (e.g. cows or individuals in poorer body condition) may favour energy-minimizing strategies. While sex-specific migratory strategies have not been reported for SRWs, sex and reproductive status influence migration in other baleen whales (e.g. [25,28]). To investigate these hypotheses, we fitted generalized linear models (GLMs) in the R *stats* package (v. 4.2.1) [57]. The binary response variable was migration route (L vs W/NW), and predictor variables included departure timing, sex, body length (hereafter length) and body condition. We created a proxy variable ‘age class’, classifying whales <12 m in length as ‘young’ and ≥12 m as ‘old’ [55]. Each predictor variable was tested in separate GLMs, with a binomial distribution and logit link function. We also built GLMs to test departure date (day-of-year) as the response variable, using a Gaussian distribution.

We compared departure and arrival dates (day-of-year), migration duration (days between departure and arrival) and travel speed between the L-shaped and W/NW outbound migration routes. Travel speed was calculated as the distance between consecutive SSM locations divided by time elapsed between them. Distances were computed using the *distanceTrack* function from the *argosfilter* package (v. 0.70) [60], and time intervals were derived from timestamp differences between successive locations. We excluded unrealistic positions with extremely high-speed values (>21 km h^–1^) [61], which exceed biologically plausible sustained swimming speeds for right whales (e.g. [61,62]) and are typically indicative of large location error associated with satellite telemetry. To account for temporal autocorrelation in consecutive speed measurements from the same individual, we compared travel speeds between routes using a linear mixed-effects model with whale tag ID as a random effect and an AR(1) autocorrelation structure (*nlme* package, v. 3.1-157) [63]. We also calculated mean speed per whale and compared these summaries between routes using Welch's *t*-test and visualized them with box plots. Tracks terminating before whales clearly reached the feeding grounds were excluded from the analyses of arrival date and migration duration but were retained for the speed analysis.

Most whales were tracked for only one outbound migration, and, accordingly, the above analyses were restricted to the first outbound migration. For whales whose tags provided data for a second outbound migration (*n* = 5), the tracks were examined qualitatively to map and compare route choice between years, as the small sample size precluded robust statistical analysis. While SSM data were used for all other analyses, raw data supplemented the dataset for one individual (tag ID: PTT215258) when comparing route choice between years, as gaps in the SSM output would have omitted substantial track details.

### (c) Cues used during migration

We hypothesized that SRW movements during migration were associated with one or more cues identified in the literature to influence whale migration across different spatial scales, including geomagnetic features and oceanographic gradients [17,21], bathymetric features (e.g. [64]) and the Antarctic sea ice edge. We tested this by overlaying environmental covariate data layers with whale tracks and extracting covariate values at each whale location. We mapped the overlaid environmental covariate data and plotted the extracted values to identify potential associations with both the first outbound (by turn phase for the L-shaped route) and inbound migration routes separately. The environmental data layers included ocean current (flow) velocity, bathymetry, geomagnetic inclination and sea ice extent (i.e. edge). Ocean current velocity data were derived from satellite altimetry provided by the Copernicus Marine Service [65] (0.125° resolution). For mapping of overlaid whale tracks relative to currents, monthly data were averaged across the outbound migration months corresponding to the timing of the L-turn (August–September 2020 and July–August 2021 and 2022) and across inbound migration months (March 2021 and January–February 2022 and 2023). To extract current velocity at individual whale locations, daily current velocity data from the same source were used. Bathymetry was derived from General Bathymetric Chart of the Oceans gridded global bathymetry data at 15 arcsecond resolution (https://www.gebco.net/data-products/gridded-bathymetry-data/gebco2023-grid). Geomagnetic inclination data were obtained from the World Magnetic Model (https://www.ncei.noaa.gov/products/world-magnetic-model). Vector shapefiles were downloaded for overlaying and mapping, and geomagnetic values were extracted to individual whale locations using the *wmm* R package (v. 1.1.1) [66]. Mean monthly sea ice extent shapefiles for mapping were sourced from the National Snow and Ice Data Center (NSIDC; https://noaadata.apps.nsidc.org/NOAA/G02135/south/monthly/). Additionally, distance from each whale location to the ice edge was calculated as the shortest great circle distance (*spDistsN1* function in the *sp* package, v. 2.2-0) [67] to the 15% ice concentration contour (specifically, the most complex contour line generated by the *rasterToContour* function in the *raster* package, v. 3.6-3) [68], using daily NSIDC SMMR-SSM/I sea ice concentration data at 25 km resolution.

## 3. Results

### (a) Migration route and timing

Sufficient data were obtained from 21 individuals to capture migration (supplementary material, figure S2) and categorize outbound migration route as L-shaped (*n* = 11) or W/NW (*n* = 10; figure 1; supplementary material, table S1), referred to as ‘cohorts’ hereafter. Across all three years, the L-cohort left earlier and travelled slower but arrived at the feeding grounds at the same time as the W/NW cohort (figure 1). Specifically, the L-cohort left one month earlier (mean day-of-year = 208, standard deviation (s.d.) = 16, median = 202, range = 189–234; figure 2A) than the W/NW cohort (mean = 235, s.d. = 28, median = 241, range = 189–268; *W* = 23, *p* = 0.026). The W/NW cohort reached the feeding grounds in significantly fewer days (mean = 42, s.d. = 8, median = 41, range = 34–56; figure 2B) compared with the L-cohort (mean = 64, s.d. = 20, median = 62, range = 40–95; *W* = 50.5, *p* = 0.016). The mean travel speed of the W/NW cohort was 3.97 km h^–1^ (s.d. = 1.92, median = 3.98, range = 0.05–18.2; figure 2C) and that of the L-cohort was 3.63 km h^–1^ (s.d. = 1.76, median = 3.59, range = 0.02–17.9). The difference in travel speed was statistically significant when assessed using a linear mixed-effects model accounting for temporal autocorrelation (*t* = 3.848, *p* = 0.001, AR(1) *ϕ* = 0.77) but not when comparing mean speeds per individual whale (Welch's *t*-test: *t* = –0.985, *p* = 0.340). Arrival at the feeding grounds was captured for 16 of the 21 whales, with no significant difference in arrival time (*W* = 23, *p* = 0.38) between the L-cohort (mean day-of-year = 274, s.d. = 15; median = 277, range = 246–289, *n* = 8; figure 2D) and the W/NW cohort (mean = 282, s.d. = 27, median = 288, range = 243–313, *n* = 8).

**Figure 1.**
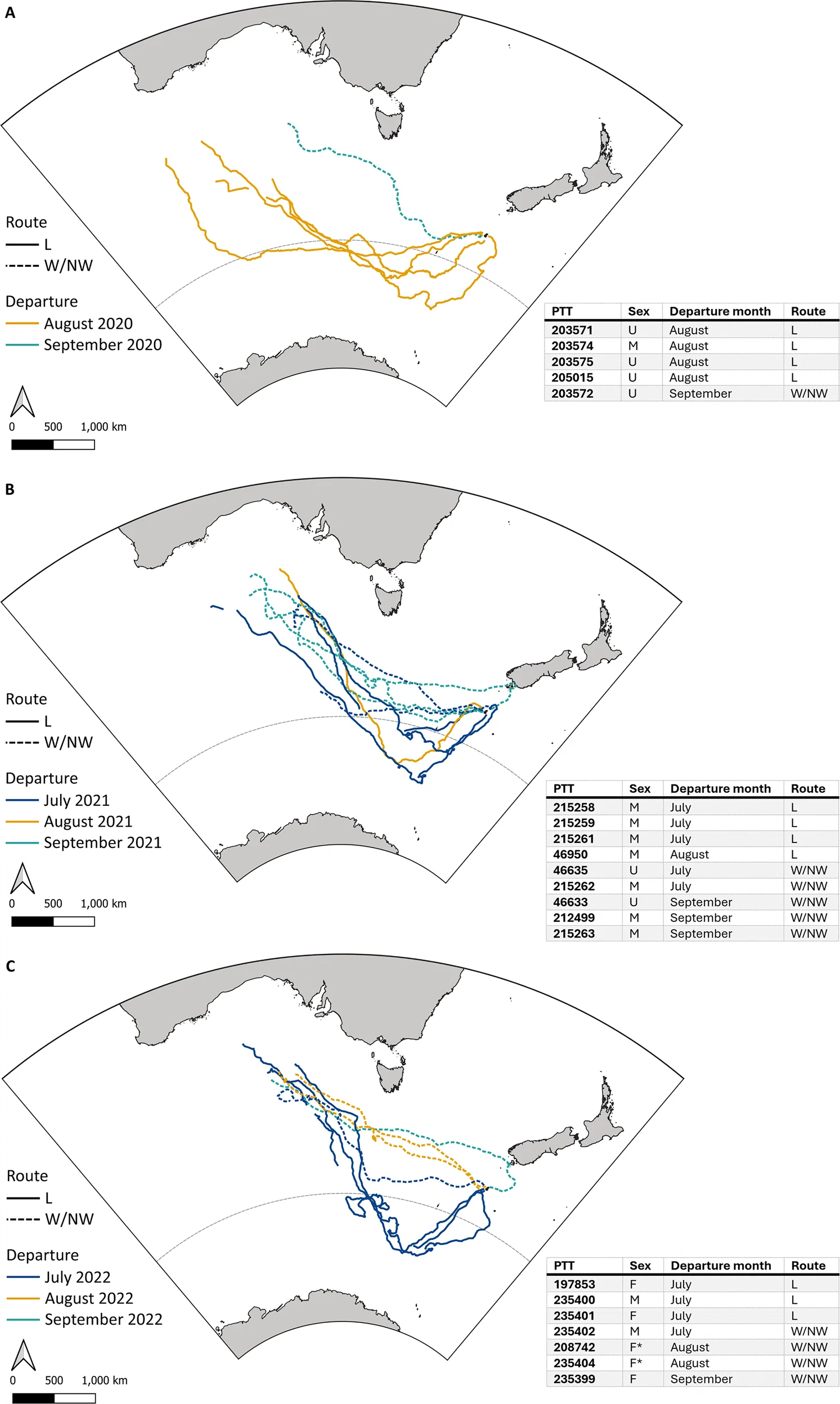
Migration routes of southern right whales in relation to departure timing. Lines represent the first outbound migration, colour-coded by departure month. Whales tagged in (A) 2020, (B) 2021 and (C) 2022. Grey dotted line indicates 55° S latitude, delineating outbound migration routes: L-shaped (L) and W/NW. Tables provide the sex (male, female and unknown), departure month and route for each individual whale (PTT). *A cow–calf pair.

**Figure 2.**
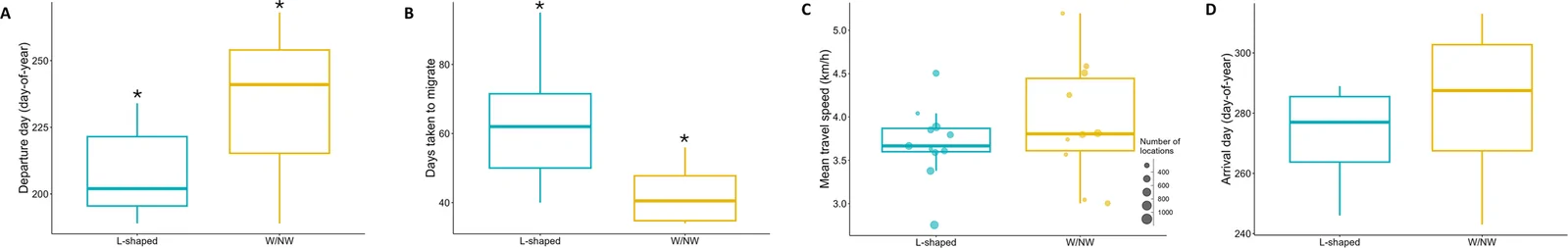
Summaries of (A) departure date, (B) migration duration (days), (C) travel speed (km h^–1^) and (D) arrival date for first outbound migrations of southern right whales using the L-shaped or W/NW route. Asterisks indicate significant differences. Departure dates (A) could be determined for all 21 whales with outbound tracks (L-shaped, *n* = 11; W/NW, *n* = 10). Plots (B) and (D) are based on *n* = 8 whales per migration route, as tracks for some whales ended too early to confirm arrival on foraging ground. (C) Mean travel speed per whale (including those whose tracks terminated before reaching the feeding grounds); the size of jittered points indicates the number of locations used to calculate each whale's mean speed. Box plots of speed measurements for all locations are provided in supplementary material, figure s3.

GLMs indicated that departure timing was the only significant predictor of outbound migration route (departure date as day-of-year: *p* = 0.028, departure month as a continuous numeric variable: *p* = 0.066; supplementary material, table S2). Outbound migration route was not significantly associated with sex, body length, age class or body condition. When treating departure date (day-of-year) as the response variable, ‘route choice’ was the only significant predictor (*p* = 0.012). Information on sex was missing for six whales, and length (and thereby age class and body condition) was missing for five whales (supplementary material, table S1), owing to either unsuccessful biopsy sampling or incomplete UAV image acquisition. The effect of reproductive status (unaccompanied adult vs cow) on departure date could not be assessed owing to the small sample size (*n* = 2 cows).

Five SRWs transmitted sufficient data to evaluate repeatability in outbound routes across two migration seasons. Of these, three whales (two males, one female) followed the same route in both years (the L-shaped route, figure 3). In contrast, two whales used the W/NW route in the first year and the L-shaped route in the second year (figure 3). One of these was a male, while the other was a female accompanied by a calf in the first year and presumably a female without a calf in the second year.

**Figure 3.**
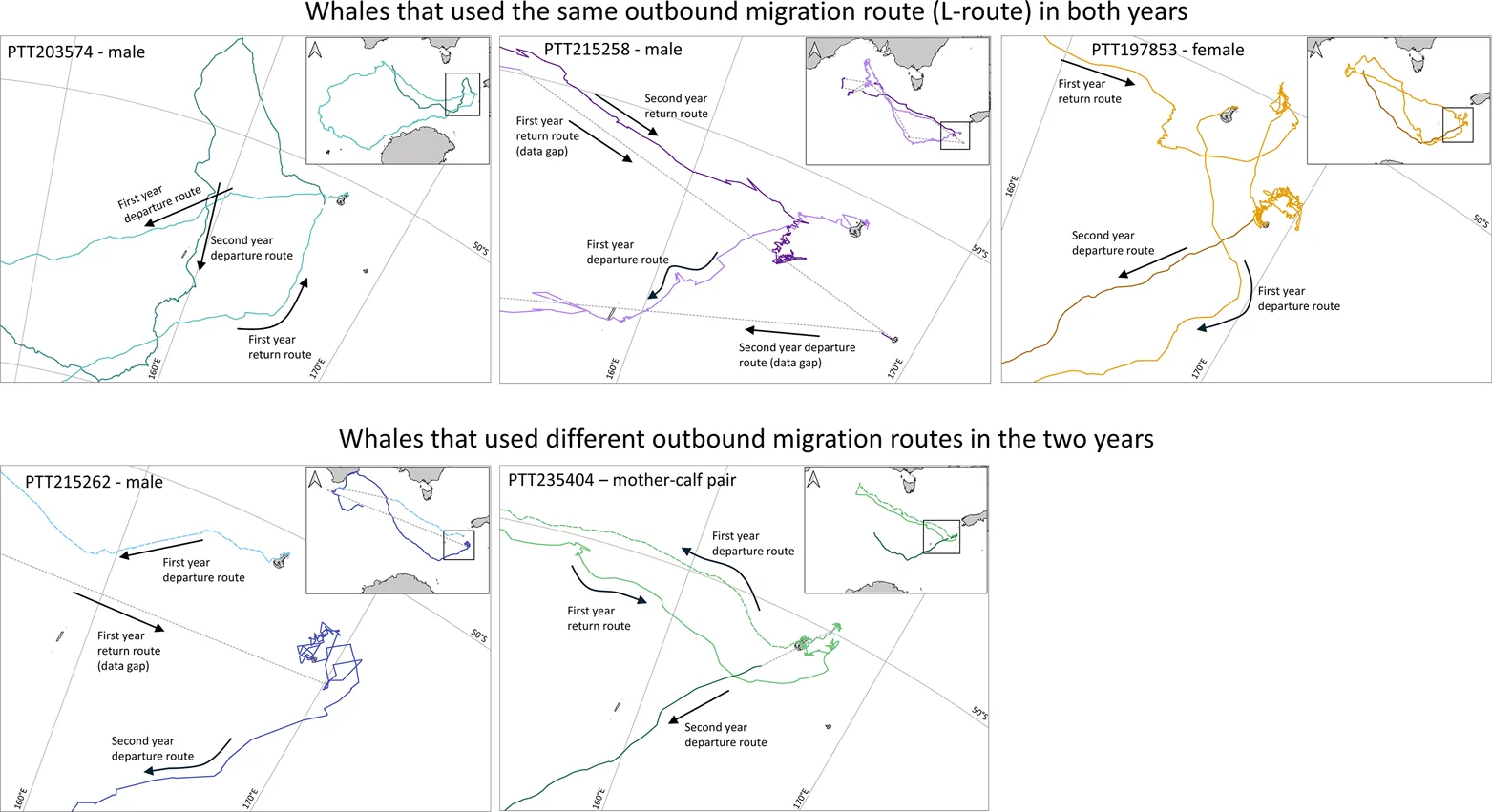
Satellite tracks of southern right whales with two outbound migrations. Lighter and darker shades indicate the first- and second years’ migrations, respectively, with dashed track lines indicating W/NW outbound migration route. L-shaped route outbound and return migrations are shown using solid track lines. Tracks were derived from state-space model locations, except for PTT215258, where data gaps required the use of raw locations. Dotted grey track lines indicate gaps in tracking data.

### (b) Cues used during migration

The L-shaped route cohort displayed a pronounced northward turn, giving the track its characteristic shape. The turn location generally coincided with the southern edge of the Antarctic Circumpolar Current (ACC, indicated by ocean current velocity; figure 4A) and variation in bathymetry (figure 4B), with whales turning near and following an area of shallower bathymetry, which topographically influences the position of the ACC. The turn location also coincided with patterns in magnetic inclination, with most whales turning in areas where magnetic inclination ranged between –84 and –82 (figure 4C; supplementary material, figure S4). The turn was less closely aligned with the ice edge; only one whale turned within 1 km of the edge, while others were 13–481 km away (figure 4D; supplementary material, figures S1 and S5). At these locations, whales altered travel direction, shifting from a predominantly southwest-oriented path to a northwest trajectory (figure 4D). In contrast, the tracks of the W/NW cohort showed no clear association with the environmental cues examined (figure 4A–C; supplementary material, figure S4).

**Figure 4.**
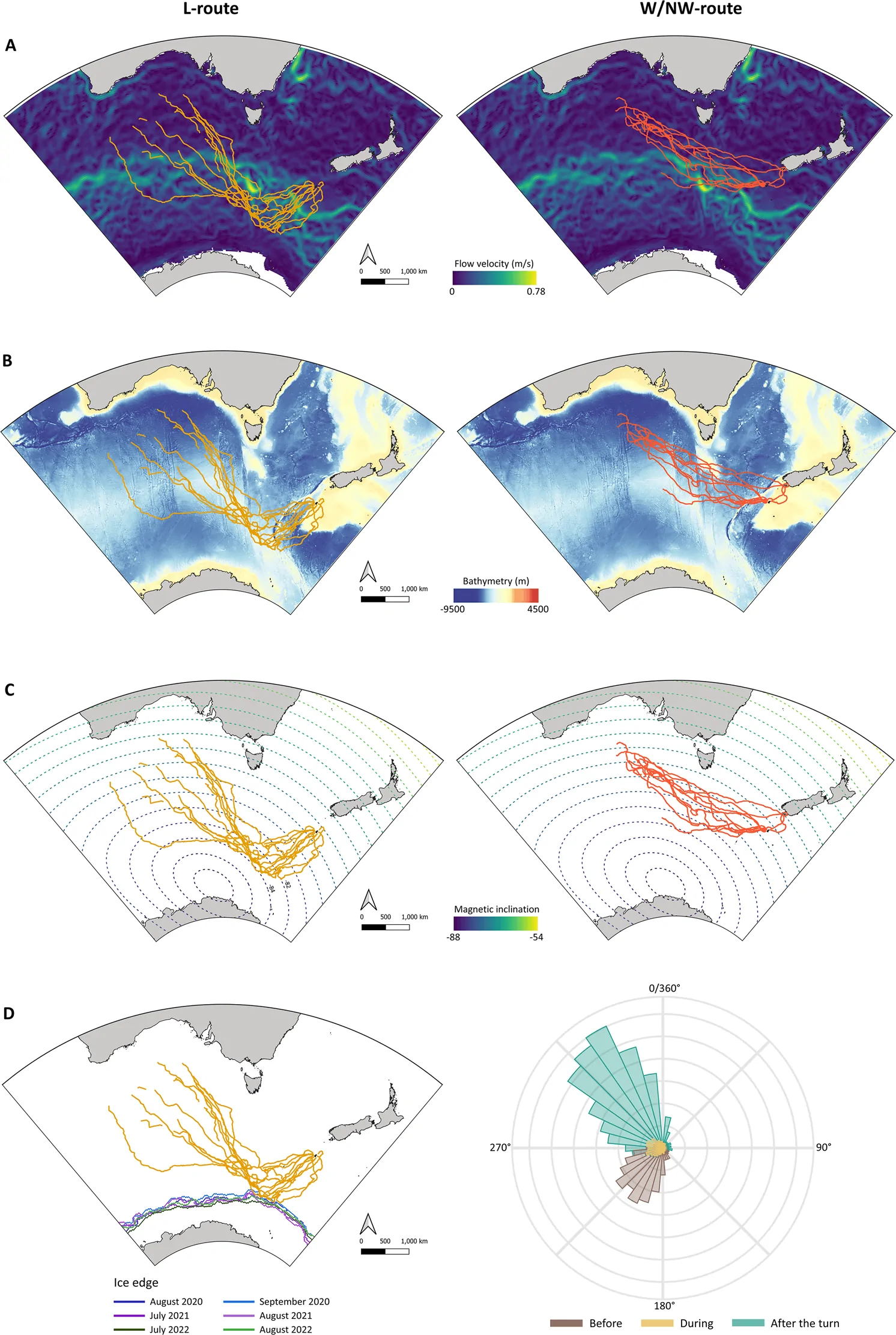
First outbound migrations of tagged southern right whales using the L-shaped or the W/NW route overlaid with (A) ocean surface current velocity, (B) bathymetry, (C) geomagnetic inclination, and (D) sea ice extent. In (D), no data are shown for whales using the W/NW route as the ice edge was not relevant given their northerly migration. Instead, the bottom panel shows the compass direction of whales using the L-route before, during and after the turn (see supplementary material, figure S1).

Eight SRWs (2020: *n* = 2; 2021: *n* = 2; 2022: *n* = 4) transmitted sufficient data to show return (inbound) migration from the feeding grounds back to the AI. Five showed strong affinity for a narrow (approx. 150 km wide) section of the ocean, with one individual using this narrow band during two return migrations (figure 5). Two additional whales had partial data showing their return towards the wintering grounds, but substantial data gaps prevented confirmation of whether they followed the narrow return pathway (figure 5). The final whale with sufficient transmission duration showed the whale returning to the AI via Antarctica (figure 3). The location of the narrow return path coincided approximately with the ACC edge and a narrow range of magnetic inclination values (between –76 and –74; figure 5; supplementary material, figure S6), but showed no obvious correlation with bathymetry (figure 5). Given the return migration occurred >1000 km north of the ice edge, the influence of the ice edge was not considered.

**Figure 5.**
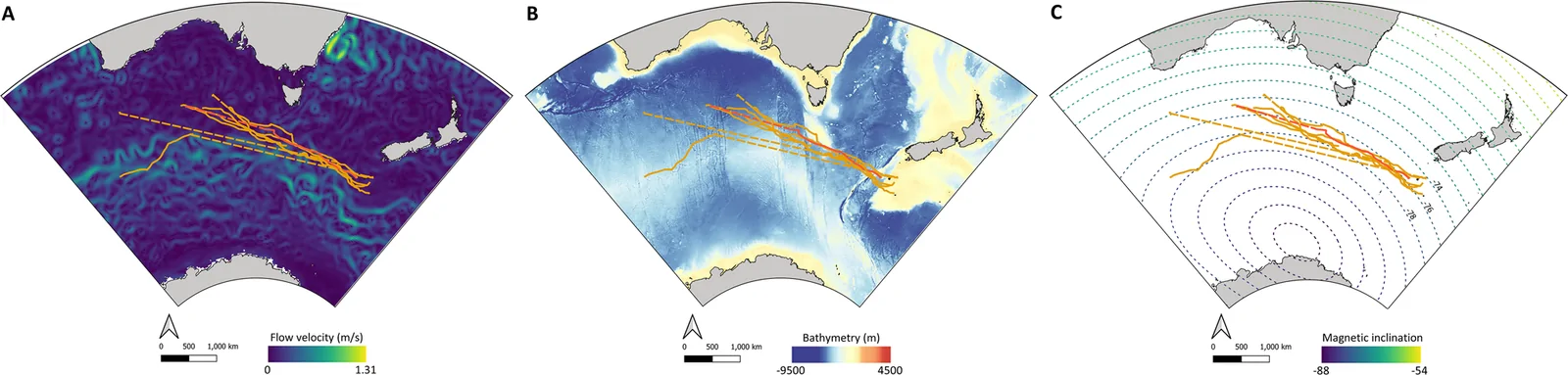
The return migration (from west to east) of tagged southern right whales (*n* = 7) overlaid with (A) ocean surface current velocity, (B) bathymetry and (C) magnetic inclination. Dashed track lines indicate gaps in tracking data. The red line indicates the second return migration by whale PTT215258.

## 4. Discussion

Understanding the drivers shaping animal migration is a fundamental ecological question and increasingly important as climate change shifts migratory phenology [69–71]. Until satellite telemetry, investigating intrinsic and extrinsic migratory cues in marine species, such as whales, was challenging. Here, we showed that SRWs migrate to a shared feeding ground via two different routes that balance time and energy constraints. Our work suggests that multiple extrinsic cues influence SRW migration across vast oceanic distances, reinforcing the idea that long-distance movements are often guided by a combination of cues (e.g. [17,72,73]).

SRWs departing the AI wintering grounds earlier were more likely to follow the longer L-route, travelling slower and taking more days to complete migration. Later-departing whales tended to use the shorter W/NW route and travelled somewhat faster, seemingly compensating for the later departure. Despite divergent routes, both groups arrived at the shared feeding grounds around the same time, with no significant differences in arrival dates. These findings are consistent with our prediction that migration in SRW reflects a trade-off between time- and energy-minimization strategies [18,19]. The convergence in arrival timing at the feeding grounds, despite the different routes, could reflect adaptation to a predictable peak in prey availability, as both strategies ensure arrival at the southern Australian feeding grounds coinciding with a seasonal peak in phytoplankton productivity [37,74].

The two routes observed in this study may reflect alternative migratory strategies that balance time and energy constraints. The longer L-shaped route is broadly consistent with an time-minimization strategy, whereas the shorter W/NW route aligns more closely with a energy-minimization strategy [18,19]. Longer migrations are often assumed costlier in terms of time, energy and mortality risk [1,75]. This could be particularly relevant for cows with calves, as a shorter, more direct route might reduce energetic costs for the calf and/or the lactating cow [76,77]. However, longer distance does not necessarily incur a negative cost. For example, migration distance and survival or reproductive success in black-backed gulls (*Larus fuscus*) were not correlated, indicating similar fitness outcomes across strategies [75], while humpback whales compensated for longer distances by swimming faster, increasing the energetic cost of transport, but completing migration sooner [30]. In aquatic systems, travel speed is a key determinant of energetic expenditure, since hydrodynamic drag, and therefore the energetic cost of swimming, increases with speed [78,79]. However, completing migration sooner may reduce cumulative costs of body maintenance and, for cows, lactation. Optimal swim speed for minimizing energy expenditure in large whales is estimated to be around 3.96 km h^–1^ [79,80]. SRWs following the W/NW route travelled slightly faster than those taking the L-shaped route, close to this optimum. However, swim speeds were broadly similar between groups, and statistical significance was inconsistent across analytical approaches, suggesting the difference may not be biologically meaningful (see also [81]). Although the differences were modest, the tendency for later-departing individuals to travel faster would be consistent with a time-minimization strategy. Overall, our results support previous findings that divergent strategies reflecting differing trade-offs can be maintained within populations, ultimately leading to similar fitness outcomes [28,82].

In many migratory taxa, including whales, departure timing and in some cases route choice have been linked to sex, age, reproductive class [7,8,25,28], body condition [29,83,84] and/or environmental factors [11,27,85]. Christiansen *et al.* [42] found that departure timing of SRW cow–calf pairs from their Australian calving grounds was primarily driven by calf size but could occur earlier if maternal body condition approached a threshold required to complete migration without falling below the survival limit (–0.40 BCI) [86]. While we found no effect of sex or body condition, this may reflect limited sample size and a bias towards tagging adult whales in good body condition. In addition, BCI was measured at the time of tagging rather than departure, with a variable time lag during which body condition may have changed [87]. However, two cow–calf pairs departed later in the season, consistent with previous findings, which would allow calves time to reach sufficient size for migration [25,27,86]. Both pairs took the W/NW route, aligning with our finding linking later departure to this pathway. For non-lactating whales, body condition is unlikely to trigger migration to feeding grounds, as studies of humpback whales show that most individuals depart breeding grounds well before exhausting their energy reserves [86], and that adult fasting endurance exceeds breeding season duration [87]. We could not examine all potential environmental predictors of departure; but, as all whales encounter similar external pre-departure conditions, environmental cues alone do not explain differences in departure timing. Instead, we suggest that departure is shaped by a combination of seasonal environmental, internal physiological and cultural cues, which, in turn, influence route selection. Importantly, if environmental cues influence both departure timing and route selection, climate-driven changes in ocean conditions (e.g. temperature, productivity) could alter these cues and modify migratory behaviour (e.g. departure or arrival time and energetic expenditure) [33,70], with potential consequences for individual fitness and population recovery.

We also found that SRW movements were associated with environmental features that could provide navigational or positional cues. During outbound migration, the distinct northward turn in L-shaped tracks coincided with multiple potential cues, including the ACC edge, bathymetry and magnetic inclination. Only one whale turned within 1 km of the ice edge, suggesting that the edge itself is not a direct cue. While infrequent, some putative feeding behaviour occurred around the L-turn, suggesting opportunistic feeding south of the polar front during late austral winter [37]. Antarctic waters typically serve as feeding grounds during the austral summer [e.g. 88]; therefore, this population appears to break the general pattern. Such supplemental feeding can be energetically rewarding (e.g. [89]) and may occur in relation to the dynamic ice edge habitat [90] and/or reduced daylight at high latitudes during austral winter, which may enhance access to vertically migrating prey [91,92]. Such conditions may have made these southern waters historically predictable feeding areas at this time of year (approx. July–September) [90], and whales may continue investigating the area for feeding opportunities before moving to their primary feeding grounds south of Australia.

During return migration, all but one whale used a narrow route, approximating the shortest path to their wintering grounds and roughly aligning with magnetic inclination patterns. This mirrors findings by Horton *et al.* [21], who reported humpback whales using a narrow migratory corridor (approx. 100 km wide) when traversing large stretches of open ocean. Many taxa use magnetic information for navigation during migration [13,16,72,83], with some species imprinting on their natal area's magnetic field during early life and later using it to guide return migrations [93,94]. For example, Manx shearwaters (*Puffinus puffinus*) appear to target specific magnetic inclination values and then follow these to their natal colony [95]. Such mechanisms could also underlie the patterns in our data, as we observed SRWs targeting magnetic inclination values between –78 and –76 and following these until close to their wintering grounds. Alternatively, the narrow return pathway could simply reflect the most direct route back to the AI. While we found associations between whale movements and several environmental features, we could not identify a single consistent cue driving route choice or turning behaviour, suggesting that SRWs may rely on multiple cues rather than a single dominant mechanism. While our data cannot establish causality between environmental features and route choice, the consistent alignment between whale tracks and environmental features provides a basis for generating further hypotheses about the sensory mechanisms underpinning SRW migration.

Beyond environmental cues, individual variability, culture and social learning can shape migratory behaviour [96]. Despite our small sample size, three individuals used the same outbound route in consecutive years, while two switched routes, suggesting some plasticity. Flexibility in route choice could reflect responses to extrinsic (e.g. resources/environmental conditions) or intrinsic factors (e.g. life-history demands). For example, studies on long-lived seasonal migrants show that individuals may adjust their migratory behaviour (e.g. route/destination) in response to changing environmental conditions, such as rising temperatures (e.g. [97]). In contrast, research on humpback whales in the South Pacific showed that individuals of different reproductive classes (cows vs adults without calves) used different migratory routes between breeding and feeding grounds (e.g. [28]). Intriguingly, one SRW in our study that changed route was a cow with a calf in the first year of tracking, and presumably without a calf the following year, suggesting reproductive status may influence route choice in SRWs. The change of routes between years also raises broader questions about migratory culture. For example, SRWs show fidelity to maternally inherited feeding grounds [40,41]. Therefore, if a calf follows its mother along a specific route, how does it later adopt a different one? It may be that young calves learn the general destination from their mother, or they initially learn one migratory route, with additional routes acquired later through extended association with the mother or social learning from conspecifics, a phenomenon observed in many social animals [98,99]. Similar mechanisms have been proposed for birds, where migratory routes can be learned from related or non-related individuals (e.g. [9]), and for humpback whales, where stopover sites may act as hubs for horizontal transmission of knowledge (e.g. migration routes or song) [28,100]. In our dataset, whales were occasionally observed migrating near one another in space and time. For example, in 2020, two individuals travelled within a day and a few kilometres of each other for approx. 400 km (supplementary material, figure S7). Such proximity suggests that whales may, at times, be within acoustic contact range, potentially enabling cultural transmission of migratory knowledge, similar to whooping cranes (*Grus americana*) and Caspian terns (*Hydroprogne caspia*), where young individuals travel with and learn migratory routes from conspecifics [101,102].

While we interpret the two route options within a time- versus energy-minimization framework, migratory strategies are unlikely to be strictly discrete. Instead, migration represents a continuum, with individuals dynamically balancing time and energy constraints depending on their condition, environmental context and life-history stage. This flexibility may explain the coexistence of alternative routes within the population and the observed ability of individuals to switch strategies between years. Individual variability and cultural learning are considered central to climate adaptability, with greater variation enhancing population resilience [97,103]. The presence of two distinct migratory routes, individual flexibility to alternate between them and potential foraging along the L-shaped route supports the existence of multiple viable strategies within this population, which may enhance resilience and represent important adaptive capacity for this recovering population in the face of climate change.

## Supplementary Material



## Data Availability

The data and code that support the findings of this study are available on Zenodo [104]. Supplementary material is available online [105].
