# Supplementary material for: Multiple strategies and cues define baleen whale migration
Source: Proc Biol Sci. 2026 Oct 14;293(2080):20260029. doi: 10.1098/rspb.2026.0029 (PMC13678521; doi:10.1098/rspb.2026.0029)
Supplement: Supplementary file 1 [file rspb.2026.0029.zip › rspb.2026.0029/rspb20260029_si_001.docx]

# Supplementary Materials

Supplementary Materials for article: *Multiple strategies and cues define baleen whale migration*

Proceedings of the Royal Society B: Biological Sciences

*DOI: 10.1098/rspb.2026.0029*

**Authors:**

Leena Riekkola^1,^ ^§, †,*^, Xuelei Zhang^1,†^, Alice Della Penna^1,2^, Virginia Andrews-Goff^3^, Gabriela Duran^1^, Rochelle Constantine^1,2^, Simon Childerhouse^4^, Fredrik Christiansen^5^, Rosalind Cole^6^, Kimberly T. Goetz^7^, Robert Harcourt^8^, David Lundquist^6^, Catherine E. Meyer^1^, Mike Ogle^6^, Esther Stuck^1^, Alexandre N. Zerbini^7,9,10,11,12^, Emma L. Carroll^1,^*

*^†^ Riekkola and Zhang should be considered joint first authors*

*^*^ Riekkola and Carroll should be considered joint corresponding authors (*[*lrie003@aucklanduni.ac.nz*](mailto:lrie003@aucklanduni.ac.nz) *&* [*e.carroll@auckland.ac.nz*](mailto:e.carroll@auckland.ac.nz)*)*

**Affiliations:**

1. School of Biological Sciences, University of Auckland – Waipapa Taumata Rau, Auckland, Aotearoa, New Zealand
2. Institute of Marine Science, University of Auckland – Waipapa Taumata Rau, Auckland, Aotearoa, New Zealand
3. Australian Antarctic Division, Department of Climate Change, Energy, the Environment and Water, Kingston, Tasmania, Australia
4. Blue Planet Marine, Nelson, Aotearoa New Zealand
5. Marine Mammal Research, Department of Ecoscience, Aarhus University, Roskilde, Denmark
6. New Zealand Department of Conservation – Te Papa Atawhai, Wellington, Aotearoa New Zealand
7. Marine Mammal Laboratory, Alaska Fisheries Science Center, National Marine Fisheries Service, National Oceanic and Atmospheric Administration, Seattle, WA, USA
8. School of Natural Sciences, Macquarie University, Sydney, NSW, Australia
9. Cooperative Institute for Climate, Ocean & Ecosystem Studies, University of Washington, Seattle, WA, USA
10. Marine Ecology and Telemetry Research, Seabeck, WA, USA
11. Instituto Aqualie, Juiz de Fora, MG, Brazil
12. Whale Unit, Mammal Research Institute, University of Pretoria, South Africa

*§ current address: Dept. Biologia Evolutiva, Ecologia i Ciències Ambientals, Universitat de Barcelona, Spain*

Supplementary Information – Supplementary Methods

Photogrammetry images were obtained using unmanned aerial vehicles (UAVs): a DJI Inspire 1 Pro fitted with a Zenmuse X5 camera and a DJI Mavic 2 Pro carrying a Hasselblad L1D-20c camera. UAVs were flown at altitudes of 14–32 m and recorded zenithal images of the whales at or near the surface and of rigid inflatable boats (RIBs) to provide reference images, enabling conversion of pixel dimensions into absolute size (m) [52]. For each whale, a single best image was retained, preferably one showing the RIB to minimize uncertainty in scale conversion. Only high- or medium-scored photographs with adequate focus and body posture were used [53]. We extracted total body length and body widths at 5% intervals using a semi-automated R script [52]. Reference-based conversion factors were calculated from the RIB images and logarithmically adjusted (β= 0.01) [54] to reduce size-related bias. For each width measurement, corresponding body height (dorsoventral distance) was estimated using an established height-to-width ratio for SRWs [55]. Body volume was calculated using the segmented elliptical model [55], based on each whale’s length, width and height measurements. We estimated body condition index (BCI) for each whale as the proportional difference between the observed (measured) and expected body volume [53]. Expected body volume (the mean volume for a given length) was derived from the log–log relationship between body volume and length established for SRWs from a healthy, growing Australian population [56]. Positive BCI values indicate individuals with greater-than-expected body volume for their length, while negative values denote poorer condition (e.g. BCI of 0.2 and –0.2 correspond to body volumes 20% above and 20% below the expected value, respectively).

*See Reference list in the main text for the citations.*

***Supplementary Table S1.*** *Dataset summary of migrating southern right whales. PTT = unique satellite tag ID for each whale; Sex = genetic sex (F = female, M= male, U = unknown); Reproductive class is either cow (with a calf) or an adult (without a calf); Outbound route = the route taken during first outbound migration, either west/northwest (W/NW) of ‘L-shaped’ route via high latitudes; Departure date and month refer to departure from the Auckland Islands wintering grounds; Reached feeding grounds = whether the track was long enough to define arrival at feeding grounds (Y = yes, N = No); Arrival date and month refer to arrival at the summer feeding grounds; Age class = category based on length: “young” if length <12 m, old if ≥12 m; BCI = body condition index. NA = measurements were not available for these individuals. A machine-readable version of the table available on Zenodo (doi.org/10.5281/zenodo.20749114).*

| PTT | Tagging year | Sex | Reproductive class | Outbound route | Departure month | Departure date (dd/mm/yyyy) | Departure date  (day-of-year) | Reached feeding grounds | Arrival month | Arrival day (dd/mm/yyyy) | Arrival day  (day-of-year) | Days taken to migrate | Length (m) | Age class | BCI |
| --- | --- | --- | --- | --- | --- | --- | --- | --- | --- | --- | --- | --- | --- | --- | --- |
| 46633 | 2021 | U | adult | W/NW | 9 | 13/09/2021 | 255 | Y | 11 | 2/11/2021 | 305 | 50 | NA | NA | NA |
| 46635 | 2021 | U | adult | W/NW | 7 | 29/07/2021 | 209 | Y | 9 | 1/09/2021 | 243 | 34 | NA | NA | NA |
| 46950 | 2021 | M | adult | L | 8 | 1/08/2021 | 212 | Y | 9 | 22/09/2021 | 264 | 52 | 9.54 | young | 0.16 |
| 197853 | 2022 | F | adult | L | 7 | 14/07/2022 | 194 | Y | 10 | 17/10/2022 | 289 | 95 | 12.30 | old | 0.07 |
| 203571 | 2020 | U | adult | L | 8 | 16/08/2020 | 228 | Y | 10 | 14/10/2020 | 287 | 59 | 11.27 | young | 0.07 |
| 203572 | 2020 | U | adult | W/NW | 9 | 25/09/2020 | 268 | Y | 10 | 29/10/2020 | 302 | 34 | 13.55 | old | -0.11 |
| 203574 | 2020 | M | adult | L | 8 | 22/08/2020 | 234 | Y | 10 | 1/10/2020 | 274 | 40 | 15.29 | old | -0.29 |
| 203575 | 2020 | U | adult | L | 8 | 8/08/2020 | 220 | Y | 10 | 12/10/2020 | 285 | 65 | NA | NA | NA |
| 205015 | 2020 | U | adult | L | 8 | 11/08/2020 | 223 | N | 10 | 5/10/2020 | 278 | 55 | NA | NA | NA |
| 208742 | 2022 | F | cow | W/NW | 8 | 29/08/2022 | 240 | Y | 10 | 3/10/2022 | 275 | 35 | 11.74 | young | 0.05 |
| 212499 | 2021 | M | adult | W/NW | 9 | 9/09/2021 | 251 | Y | 10 | 19/10/2021 | 291 | 40 | 14.40 | old | 0.02 |
| 215258 | 2021 | M | adult | L | 7 | 22/07/2021 | 202 | Y | 9 | 4/09/2021 | 246 | 44 | 11.64 | young | -0.04 |
| 215259 | 2021 | M | adult | L | 7 | 17/07/2021 | 197 | Y | 9 | 21/09/2021 | 263 | 66 | NA | NA | NA |
| 215261 | 2021 | M | adult | L | 7 | 20/07/2021 | 200 | N | 9 | 13/09/2021 | 255 | 55 | 11.02 | young | -0.12 |
| 215262 | 2021 | M | adult | W/NW | 7 | 19/07/2021 | 199 | N | 8 | 22/08/2021 | 233 | 34 | NA | NA | NA |
| 215263 | 2021 | M | adult | W/NW | 9 | 24/09/2021 | 266 | Y | 11 | 10/11/2021 | 313 | 47 | 10.95 | young | 0.17 |
| 235399 | 2022 | F | adult | W/NW | 8 | 31/08/2022 | 242 | Y | 10 | 11/10/2022 | 284 | 41 | 11.64 | young | 0.06 |
| 235400 | 2022 | M | adult | L | 7 | 9/07/2022 | 189 | N | 9 | 13/09/2022 | 255 | 66 | 13.89 | old | -0.15 |
| 235401 | 2022 | F | adult | L | 7 | 12/07/2022 | 192 | Y | 10 | 8/10/2022 | 280 | 88 | 11.13 | young | 0.07 |
| 235402 | 2022 | M | adult | W/NW | 7 | 9/07/2022 | 189 | Y | 9 | 3/09/2022 | 245 | 56 | 13.42 | old | -0.14 |
| 235404 | 2022 | F | cow | W/NW | 8 | 23/08/2022 | 234 | N | 10 | 18/10/2022 | 290 | 56 | 15.96 | old | 0.07 |

***Supplementary Table S2.*** *Generalised Linear Model results and parameter estimates. The binary response variable for each univariate model was migration route (‘L-shaped’ or W/NW). The covariates with a significant p-value are highlighted in bold. For more information about the covariates see Supplementary Table S1.*

| Model | Parameter | Value | Std. Error | z-value | *p* |
| --- | --- | --- | --- | --- | --- |
| ~Departure day  (as day-of-year) | Intercept | -11.89 | 5.36 | -2.22 | 0.027 |
|  | **Departure day** | 0.05 | 0.02 | 2.20 | **0.028** |
| ~Departure month | Intercept | -10.18 | 5.49 | -1.85 | 0.064 |
|  | Departure month | 1.30 | 0.71 | 1.84 | 0.066 |
| ~Sex | Intercept | 0.41 | 0.91 | 0.44 | 0.657 |
|  | Sex - male | -0.81 | 1.12 | -0.73 | 0.468 |
|  | Sex - unknown | -0.41 | 1.22 | -0.33 | 0.741 |
| ~Sex  (excluding unknown sex) | Intercept | 0.41 | 0.91 | 0.44 | 0.657 |
|  | Sex - male | -0.81 | 1.12 | -0.73 | 0.468 |
| ~Length | Intercept | -4.86 | 4.13 | -1.18 | 0.240 |
|  | Length | 0.38 | 0.33 | 1.15 | 0.249 |
| ~ Age class  (length category) | Intercept | 0.29 | 0.76 | 0.38 | 0.706 |
|  | Young | -0.80 | 1.06 | -0.76 | 0.450 |
| ~Body condition | Intercept | -0.11 | 0.53 | -0.22 | 0.828 |
|  | Body Condition | 3.17 | 4.38 | 0.73 | 0.468 |

***Supplementary Figure S1.*** *Map illustrating how locations along outbound L-route tracks were classified as occurring before (brown), during (red), or after (yellow) the L-turn. Blue, purple and green lines show the mean monthly sea ice extent during months that had locations classified as “during the turn”. The accompanying table provides date ranges of locations classified as “during the turn” for each tagged whale (PTT). Note that due to a data gap the exact location and timing of the turn could not be identified for PTT215258. For PTT203574, no clear “during the turn” phase was observed, so a single pivot point was used as the turn location. Inset maps provide example tracks: whale PTT197853 demonstrates that the turn was not always sharp, resulting in a distinct “during the turn” phase, whereas PTT203574 shows a case without a clear “during the turn” phase. Whale PTT197853 performed the turn in August 2022, and the corresponding monthly mean sea ice extent is shown.*

***Supplementary Figure S2.*** *Migratory tracks of tagged southern right whales (n = 21), categorized by tagging years: 2020 (green, n = 5), 2021 (purple, n = 8) and 2022 (orange, n = 7).*

**Supplementary Figure S3.** Boxplot s*ummaries of travel speed (km/h) of all locations for first outbound migrations of southern right whales using either the ‘L-shaped’ or ‘W/NW’ migration route. All tracks (‘L-shaped’ route, n=11 whales; W/NW route, n=10 whales), including those that terminated before reaching the feeding grounds, were used to calculate travel speeds.*

***Supplementary Figure S4.*** *Density plots of environmental covariates at each southern right whale location during the first outbound migration along either the ‘L-shaped’ or west/northwest (W/NW) route. Panels show distributions of (A) ocean current flow velocity (m/s), (B) bathymetry (m), (C) magnetic inclination, and (D) distance to the ice edge (km). Whales migrating along the ‘L-shaped’ route are split into three turn phases: before, during, and after the L-turn (see main Methods and Supplementary Figure S1 for details). For maps of outbound migrations overlaid with the environmental covariates, see main Figure 4.*

***Supplementary Figure S5.*** *Relationship between distance from the turn and distance from the ice edge for whales using the ‘L-shaped’ outbound migration route. The x-axis shows distance from the turn, with 0 representing locations classified as “during the turn”, negative values indicating positions “before the turn”, and positive values indicating positions “after the turn” (see Supplementary Figure S1). The y-axis shows the distance from the ice edge (daily 15% ice contour) at the corresponding locations. Each point represents a whale location and is colour-coded by whale tag ID (PTT). The table shows the minimum and maximum distances from the ice edge for locations classified as “during the turn” for each tagged whale. Note that due to a data gap the exact location of, and distance from, the turn could not be identified for PTT215258. For PTT203574, no clear “during the turn” phase was observed, so a single pivot point was used as the turn location (see Supplementary Figure S1), resulting in identical minimum and maximum distances.*

***Supplementary Figure S6.*** *Density plots of environmental covariates at each southern right whale location during inbound migrations. Panels show distributions of (A) ocean current flow velocity (m/s), (B) bathymetry (m), and (C) magnetic inclination. For maps of inbound migrations overlaid with the environmental covariates, see main Figure 5.*

**A**

**B**

**C**

***Supplementary Figure S7.*** *Tracks for southern right whales satellite tagged in (A) 2020, (B) 2021, and (C) 2022, illustrating examples where individual whales were within a few days and within a few kilometres of each other. In 2022, the example of PTT235401 and PTT235404 highlights that whales could also travel through remarkably similar locations several days apart.*
